# Editorial: Exploring the role of plant phytochemical diversity in biodiversity and beyond

**DOI:** 10.3389/fpls.2026.1841664

**Published:** 2026-05-01

**Authors:** Arbi Guetat

**Affiliations:** Department of Biological Sciences, College of Science, Northern Border University, Arar, Saudi Arabia

**Keywords:** biodiversity, biosynthetic pathways, functional diversity, metabolomics, multitrophic interactions, phytochemical diversity, plant secondary metabolites

Biodiversity, encompassing the variety of life across all sources, forms the foundation of Earth’s ecosystems. It includes genetic variation within species, differences among species, and the diversity of ecological communities and habitats (World Health Organization, 2026). Genetic diversity refers to the variety of alleles and genotypes present in a given group under study (population, species, or group of species) (Frankham et al., 2010). Species diversity is the variety of different species within a habitat, region, or the entire planet (Kiester, 2013). Ecosystem diversity encompasses the range of habitats, biological communities, and ecological processes present across the biosphere (Australian Government Department of Agriculture, Water and the Environment, 2003). It includes the interactions between living organisms (biotic factors) and their non-living environment (abiotic factors). Moreover, the concept of species, as established by Ernest Mayer as “the basic category of biological classification,” is still the fundamental unit of study in the field of Biology (de Queiroz et al., 2005). Chemodiversity can be defined as all forms of chemical diversity produced by living organisms, including Phytochemical Diversity (plant secondary metabolites); Mycochemical Diversity (Fungi secondary metabolites); the biosynthetic diversity of animals, Bacterial and Archaeal metabolic diversity, and metabolic responses induced by exogenous particles, such as viruses. Phytochemical diversity is a key component of functional diversity (Richards et al., 2015). By definition, functional diversity encompasses the traits and roles within species that shape ecosystem dynamics, stability, productivity, and nutrient regulation (Tilman, 2001). Phytochemical diversity in plants is extremely important for key evolutionary and adaptive mechanisms. However, specialized metabolites, such as Alkaloids, Terpenoids, and Phenolics, which mediate vital and key evolutionary processes, are invisible dimensions of biodiversity (Wetzel and Whitehead, 2020; Defossez et al., 2021). These metabolites are involved in critical ecological interactions such as plant defense against herbivores and pathogens, allelopathy, and responses to abiotic stressors, thereby influencing community dynamics and ecosystem functioning (Petrén et al., 2023; Salam et al., 2023). Phytochemical diversity mediates the majority of multitrophic interactions, affecting herbivore diets, predator–prey relationships, and parasite defenses (Abdala-Roberts and Moreira, 2024). The functional dimensions of biological variation can be predicted across landscapes and appear to be linked to environmental gradients, making them analogous to classical taxonomic biodiversity (Wetzel and Whitehead, 2020; Defossez et al., 2021). Furthermore, actual advances in metabolomics and quantitative methods underpin the importance of integrating biochemical structures and biosynthetic pathways into biodiversity and chemodiversity assessments (Defossez et al., 2021). Considering these factors, phytochemical diversity functions as an invisible driver of ecological complexity that enriches our understanding of how biological variation influences ecosystem resilience, species interactions, and conservation priorities.

A recent Research Topic in “Frontiers in Plant Science” explored pioneering research aimed at developing Plant Metabolism and Chemodiversity with the focus on “*Exploring the Role of Plant Phytochemical Diversity in Biodiversity and Beyond*”.

This Research Topic features five original research articles and one comprehensive review that collectively advance our understanding of the functional dimensions of biological variation.

This Research Topic outlined the roles of metabolomics and chemodiversity and their applications in the fields of agriculture, medicine, and the sustainable valorization of phytoresources. Taken together, these contributions explored the importance of the genetic pool, environmental impact, and developmental factors that influence chemical composition and bioactivity, along with the application of plant-derived compounds across a varied range of species. Several studies highlighted the phytotoxicity and antifungal activity of plant extracts and essential oils from different plant species. The phytochemicals investigated from *Eucalyptus parvula*, *Melaleuca alternifolia*, and various medicinal and aromatic plants could constitute eco-friendly alternatives to synthetic pesticides. On the other hand, to overcome limitations within the *Ferula* complex taxa, research integrated phylogenomics tools with a metabolomics approach. Other studies focused on *Forsythia suspensa*, exposing how changes in metabolites during developmental stages or across environments influence quality traits; this included phenolic acids in *Forsythia suspensa* and volatile phenols in grape species. Furthermore, studies focusing on *Tinospora* and *Ligusticum chuanxiong* combined the impact of environmental factors, chemical profiling, and pharmacological investigations to optimize cultivation and harvesting strategies. Moreover, to investigate the nutraceutical potential of citrus peels, a comprehensive profiling approach was conducted through the valorization of these agro-industrial by-products via genetic profiling, GC-MS, and nutritional analysis. Finally, a synthesis analysis of *Eucalyptus* phenolics positioned the phytochemicals of this genus as strong candidates for innovation in the era of synthetic biology. Together, these contributions underpin the importance of integrating genetic, metabolomic, and ecological approaches to unlock the extraordinary potential of plant diversity as a renewable and sustainable source of multipurpose phytochemicals.

## References

[B1] Abdala-Roberts L. Moreira X. (2024). Effects of phytochemical diversity on multitrophic interactions. Curr Opin Insect Sci. 64, 101228. doi: 10.1016/j.cois.2024.101228, PMID: 38944275

[B2] Australian Government Department of Agriculture, Water and the Environment (2003). Ecosystem diversity: Conservation of biological diversity. Available online at: https://www.agriculture.gov.au/sites/default/files/documents/SOFR_2003_Chapter_1_Conservation_of_biological_diversity.pdf.

[B3] Defossez E. Pitteloud C. Descombes P. Glauser G. Allard P. M. Walker T. W. N. . (2021). Spatial and evolutionary predictability of phytochemical diversity. PNAS 118, e2013344118. doi: 10.1073/pnas.2013344118. PMID: 33431671 PMC7826413

[B4] De Queiroz K. (2005). Ernst Mayr and the modern concept of species. Proceedings of the National Academy of Sciences 102(suppl_1), 6600–6607. doi: 10.1073/pnas.0502030102. PMID: . Epub 2005 Apr 25. PMID: 15851674; PMCID: PMC1131873. 15851674 PMC1131873

[B5] Frankham R. Ballou J. D. Briscoe D. A. (2010). Introduction to Conservation Genetics (2nd ed.). Cambridge, UK: Cambridge University Press.

[B6] Kiester A. R. (2013). “ Species diversity, overview,” in Encyclopedia of Biodiversity (Second Edition). Ed. Levin S. A. ( Academic Press), 706–714. doi: 10.1016/B978-0-12-384719-5.00133-7, PMID:

[B7] Petrén H. Köllne T. G. Junker R. R. (2023). Quantifying chemodiversity considering biochemical and structural properties of compounds with the R package chemodiv. New Phytol. 237 (6), 2478–2492. doi: 10.1111/nph.18685, PMID: 36527232

[B8] Richards L. A. Dyer L. A. Forister M. L. Smilanich A. M. Dodson C. D. Leonard M. D. . (2015). Phytochemical diversity drives plant–insect community diversity. Proc. Natl. Acad. Sci. 112, 10973–10978. doi: 10.1073/pnas.1504977112. PMID: 26283384 PMC4568244

[B9] Salam U. Ullah S. Tang Z. H. Elateeq A. A. Khan Y. Khan J. . (2023). Plant metabolomics: An overview of the role of primary and secondary metabolites against different environmental stress factors. Life. 13 (3), 706. doi: 10.3390/life13030706, PMID: 36983860 PMC10051737

[B10] Tilman D. (2001). Functional diversity. In Encyclopedia of Biodiversity (pp. 587–596). San Diego, CA, USA: Elsevier Inc.

[B11] Wetzel W. C. Whitehead S. R. (2020). The many dimensions of phytochemical diversity: Linking theory to practice. Ecol. Lett. 23, 16–32. doi: 10.1111/ele.13422. PMID: 31724320

[B12] World Health Organization (2026). Biodiversity. Available online at: https://www.who.int/news-room/fact-sheets/detail/biodiversity (Accessed March 27, 2026).

